# Political Beliefs and Legitimacy of Government Restrictions During the COVID-19 Pandemic

**DOI:** 10.3390/bs16050765

**Published:** 2026-05-13

**Authors:** Marek Palace, Manish Madan, Brandon May, Lee Smith, Sarah Daly, Sylvia Terbeck, Torrin Jacobson

**Affiliations:** 1 School of Psychology, Liverpool John Moores University, Liverpool L2 2QP, UK; s.terbeck@ljmu.ac.uk; 2 School of Social and Behavioral Sciences, Stockton University, Galloway, NJ 08205, USA; 3 School of Psychology, Florida Institute of Technology, Melbourne, FL 32901, USA; 4 Centre for Health Performance and Wellbeing, Anglia Ruskin University, Cambridge CB1 1PT, UK; 5 Department of Psychological Science, Saint Vincent College, Latrobe, PA 15650, USA

**Keywords:** COVID-19 pandemic, individual factors, political views

## Abstract

The current paper examines how individual/personality factors are associated with the political legitimacy of government restrictions at the onset of the COVID-19 pandemic. A total of 1262 US-based participants completed an online survey comprising several scales (predictor factors), such as the *Just World Scale*, *the Police Legitimacy Scale*, and the Authoritarianism Scale measuring aggression, submission, and conventionalism. In addition, they completed scales measuring their *Fear of COVID* and *Perceptions of Government* (outcome factors). The results suggest that those who viewed the president or federal government as most responsible had lower legitimacy scores than those who reported their governor, state government, or local official or government to be responsible. Also, those who aligned with the Republican party had the lowest mean for fear of COVID, while the highest was in the “Other” political affiliation, followed by the Democrats, who had the second highest. It also turned out that whereas one’s relationships with those who have been hospitalized or died as a result of COVID and individual risk factors for COVID were not significant variables in predicting perceptions of the federal government’s handling of the pandemic, the most significant factors were *Authoritarianism, Fear of COVID-19, (older) Age, Change in Federal Trust* and *Political Ideology*. *Fear of COVID-19* was the only significant factor predicting government legitimacy and individual decisions to engage in protection measures during the pandemic. Theoretical and practical implications are discussed.

## 1. Introduction

The SARS-CoV-2 pandemic precipitated an extraordinary expansion of governmental authority, with jurisdictions worldwide implementing non-pharmaceutical interventions (NPIs) of unprecedented scope and severity. These measures, which included mandatory lockdowns, business closures, travel restrictions, and quarantine mandates, represented the most extensive peacetime restrictions on civil liberties in modern democratic history (Gostin & Chertoff, 2021; Gostin & Wiley, 2020). In the United States, both federal and state governments invoked emergency health powers to impose these restrictions, raising questions regarding the scope, legitimacy, and effectiveness of governmental authority during public health crises. By April 2020, approximately 3.9 billion individuals globally, nearly half of the world’s population, were subject to stay-at-home orders (Gostin & Chertoff, 2021). However, public compliance with and support for these interventions varied substantially, with evidence suggesting that this variation was structured by interactions among demographic, psychological, and political factors.

### 1.1. Political Polarization and Pandemic Response

Research has demonstrated that political identity constituted the primary determinant of individuals’ pandemic-related attitudes and behaviors, frequently superseding the influence of direct experience with the virus (e.g., Collins et al., 2021; Gadarian et al., 2021; Makridis & Rothwell, 2020; Raile et al., 2024). For instance, Collins et al. (2021) analyzed data from 6383 American adults and found that political party affiliation predicted emotional distress, threat perception, support for restrictions, and evaluations of governmental response with greater magnitude than personal economic impact (i.e., job or wage loss). This finding supports theoretical frameworks emphasizing the role of political identity in information processing and attitude formation (see also Lindow et al., 2021). The observed partisan divide reflected normative value differences between political coalitions and was reinforced by divergent elite messaging regarding pandemic severity and appropriate policy responses. Democrats and Republicans specifically demonstrated diametrically opposed assessments of whether individuals and institutions had overreacted or underreacted to the viral threat, with these evaluations strongly predicted by partisan affiliation (Collins et al., 2021).

Subsequent research has replicated and extended these findings across samples and methodological approaches. Christensen et al. (2020) documented that political conservatives exhibited significantly lower perceived threat from COVID-19 and were substantially more likely to characterize media coverage as excessive during the initial phase of social distancing implementation. Hsiehchen et al. (2020) employed mobility data derived from anonymous mobile phone tracking to assess adherence to NPIs, finding that state-level partisan composition predicted compliance with movement restrictions even after controlling for infection rates and governmental policies. This behavioral divergence along partisan lines persisted despite the alleged apolitical nature of viral transmission, suggesting that political identity fundamentally shaped risk perception and behavioral responses (Hardy et al., 2021).

### 1.2. Ideology as a Multidimensional Predictor of COVID-19 Beliefs and Behaviors

While partisan affiliation represents a powerful heuristic for understanding pandemic attitudes, research examining specific ideological dimensions provides an additional theoretical lens. Gerace et al. (2022) demonstrated that support for easing COVID-19 restrictions was predicted not only by political party identification but also by degrees of social and economic conservatism, independent of demographic covariates including gender, age, ethnicity, and education. Perry et al. (2021) analyzed the role of Christian nationalism in structuring opposition to governmental restrictions, identifying both libertarian and authoritarian impulses within this belief system that supported resistance to state mandates. Adherents cited concerns regarding economic disruption and threats to individual liberty, framing pandemic restrictions as governmental overreach inconsistent with religious and political values.

Further, Peng (2022) employed a multidimensional approach to ideological measurement, simultaneously examining social dominance orientation, right-wing authoritarianism, and libertarianism as predictors of COVID-19 reactions across both convenience and nationally representative samples. Results indicated that libertarianism and social dominance orientation predicted opposition to governmental interventions, while authoritarianism demonstrated more nuanced effects contingent upon the source and framing of directives. Interestingly, this research seemed to suggest that political ideology operates through multiple psychological dimensions beyond simple left-right orientations, with distinct features differentially predicting pandemic-related cognitions and behaviors.

The apparent paradox of conservative resistance to governmental directives, given that conservative ideology typically emphasizes rule-following, loss aversion, and deference to authority (e.g., Clarke, 2017; Jost et al., 2003; Turner, 2010), has been resolved through content analysis of elite political communication. Lindow et al. (2021) analyzed semantic content from local and federal press releases alongside media coverage, demonstrating that conservative political leaders frequently framed pandemic responses in ways that undermined compliance among their constituents. This finding suggests that observed partisan differences in compliance reflect not inherent ideological incompatibility with collective action but rather the influence of elite cue-giving and partisan motivated reasoning.

Yet even this expanded ideological account does not fully explain the substantial heterogeneity in how individuals interpreted risk, evaluated governmental directives, or responded behaviorally during the pandemic. Political ideology clearly structures attitudes, but it does not operate in a psychological vacuum. To understand why individuals with similar ideological commitments nevertheless diverged sharply in their reactions, it becomes necessary to examine the role of personality and broader individual-difference variables. Specifically, individual personality characteristics have been identified as secondary but nonetheless significant predictors of pandemic-related attitudes and behaviors.

Zajenkowski et al. (2020) examined both *Five-Factor Model* traits and *Dark Triad* characteristics in relation to compliance with governmental restrictions, finding that low agreeableness and elevated Machiavellianism, psychopathy, and narcissistic rivalry predicted reduced compliance. Critically, their research demonstrated that situational perceptions accounted for greater variance in compliance than dispositional traits, consistent with theoretical perspectives emphasizing the constraining effects of strong situations on personality expression. This finding suggests that pandemic-related behaviors may be more malleable through situational interventions than through targeting ‘stable’ individual differences.

Lippold et al. (2020) conducted cross-national and longitudinal analyses of personality correlates of coronavirus fear, identifying complex interactions among personality traits, political attitudes, and sociodemographic characteristics. Their work demonstrated that while personality factors contributed to pandemic responses, these effects were frequently moderated by political orientations and situational appraisals. However, Dunnum and Neal (2025) examined the relationship between personality (specifically openness and conscientiousness) and political identity in predicting COVID-19 news consumption patterns, finding that openness predicted reduced consumption of conservative-oriented pandemic coverage but did not significantly predict liberal news use. These findings broadly suggest that information-seeking behaviors during the pandemic were structured by interactions between dispositional characteristics and political identities, and not political identities in and of themselves.

### 1.3. Fear, Emotional Responses, and Mental Health Consequences

Affective responses to the pandemic, particularly fear and anxiety, have been consistently identified as determinants of both attitudinal and behavioral outcomes (Bish & Michie, 2010; Kousoulis et al., 2021; Sharif et al., 2022; Usher et al., 2020). For example, large-scale epidemiological studies documented elevated psychological distress among American adults during the early pandemic period (e.g., Breslau et al., 2021; McGinty et al., 2020). Fitzpatrick et al. (2020) analyzed data from 10,368 respondents and found that COVID-19-related fear and perceived threat significantly predicted anxiety and depressive symptomatology, with effects moderated by social vulnerabilities and community characteristics. French et al. (2020) similarly documented elevated psychological distress during the initial pandemic phase, with mean scores suggesting widespread mental health impacts requiring policy attention.

Subsequently, Sloan et al. (2021) advanced theoretical understanding by distinguishing between personal fear regarding one’s own infection risk and altruistic fear concerning others’ wellbeing, demonstrating that both constructs were prevalent and consequential for mental health outcomes. Building on this broader effort to differentiate psychological responses, Jacobs and Burch (2021) employed Census Bureau Household Pulse Survey data to examine the temporal trajectory of anxiety from April through July 2020, documenting differential patterns across racial and ethnic groups. Their negative binomial regression analyses revealed that anxiety evolved heterogeneously across demographic categories, suggesting that pandemic-related distress was not uniformly distributed across the population.

Beyond mental health consequences, fear demonstrated significant effects on policy attitudes and behavioral compliance. Liu and Yang (2023) found that discrete emotional states, particularly fear, predicted public support for COVID-19 policy responses, with effects mediated by cultural cognition and risk perception. Vasilopoulos et al. (2023) provided particularly nuanced empirical evidence regarding the interaction between fear and governmental trust using panel data from five European democracies. Their analyses suggested that experiencing COVID-19-related fear substantially reduced the moderating effect of governmental trust on support for civil liberty restrictions. Specifically, high-fear individuals demonstrated elevated support for restrictive measures regardless of their baseline trust in government, suggesting that threat-based emotions can override typical political predispositions under crisis conditions.

However, the relationship between anxiety and political reasoning may be more nuanced than simple override effects. Mehlhaff et al. (2024) tested whether anxiety about COVID-19 illness interrupted partisan motivated reasoning, employing panel data to assess dynamic relationships between emotional states and political judgment. While motivated reasoning remained prevalent, their findings indicated that anxiety encouraged more evenhanded evaluation in specific domains, though the scope and consistency of this effect were circumscribed. Yet even these findings, which suggest limited circumstances under which anxiety can temper partisan reasoning, point toward a broader question regarding the *content* of political fear itself. To understand not just whether anxiety alters political judgment but how individuals interpret and assign meaning to that anxiety, it is necessary to examine the narratives through which fear becomes politicized. For instance, Hardy et al. (2021) employed mixed methods combining qualitative interviews with quantitative survey data to examine politicized fear, identifying both convergence and divergence in how fear manifested across partisan divides. Their research revealed that political ideology shaped not only fear intensity but also attributions of responsibility and blame for pandemic-related outcomes.

### 1.4. Governmental Legitimacy and Crisis Response

Questions of governmental legitimacy have become central to understanding compliance with pandemic restrictions and evaluations of crisis management. Indeed, Gostin and Wiley (2020) noted that emergency health powers invoked by federal and state governments to implement NPIs raised fundamental questions regarding the appropriate scope of governmental authority during public health emergencies. The federal structure of American governance complicated legitimacy assessments, as citizens often had to overcome overlapping and sometimes conflicting directives from federal, state, and local authorities, each claiming jurisdiction over different aspects of pandemic response (e.g., Coglianese, 2022).

Jacobs (2021) demonstrated that partisan identity structured attitudes toward governmental legitimacy and responsibility differently across federal levels. Democrats and Republicans assigned divergent functional responsibilities to local, state, and federal governments, fundamentally disagreeing about appropriate divisions of intergovernmental authority during the crisis. This fragmentation of legitimacy perceptions across governmental levels was further complicated by the concurrent occurrence of multiple national crises. For instance, Jipguep-Akhtar et al. (2021) examined the collision of the pandemic with protests for racial justice, noting that both crises challenged police legitimacy while simultaneously expanding law enforcement responsibilities to include public health mandate enforcement. This convergence created novel tensions regarding the appropriate scope and legitimacy of state authority.

Theoretical frameworks for understanding legitimacy during crisis conditions have emphasized the multidimensional nature of legitimacy perceptions, including assessments of procedural fairness, distributive justice, effectiveness, and lawfulness. However, most existing legitimacy research has focused on criminal justice contexts, with limited application to public health emergency responses. The extent to which traditional legitimacy dimensions operate similarly in public health versus criminal justice contexts remains an open empirical question.

### 1.5. The Current Research

Despite substantial research attention to political and psychological factors shaping pandemic responses, gaps remain in our understanding of how these variables interact to influence legitimacy perceptions and behavioral compliance in the American context. Existing literature has documented partisan divides in attitudes and identified fear as a significant motivating factor, yet several questions warrant further empirical investigation. First, the relationship between attributions of governmental responsibility for pandemic response and subsequent legitimacy judgments has received limited systematic attention. Second, the relative predictive utility of direct experience with COVID-19 (through personal risk factors or social network exposure) compared to more distal political and emotional factors remains underspecified. Third, existing research has not adequately examined how legitimacy perceptions mediate relationships between individual-level factors and health-protective behaviors across different governmental entities.

The present study addresses these gaps by examining relationships among responsibility attributions, legitimacy perceptions, and individual-level predictors, including political ideology, authoritarianism, fear of COVID-19, and direct pandemic experience. Specifically, this study tests three sets of research questions: (1) How do attributions of primary responsibility for pandemic response (federal, state, or local governmental entities) relate to legitimacy perceptions? (2) Which individual-level factors, including political ideology, authoritarianism, fear of COVID-19, personal risk status, and social network exposure, predict perceptions of governmental response and legitimacy? (3) How do legitimacy perceptions and individual-level factors influence engagement in protective health behaviors?

## 2. Methodology

The study analyzed 2020 data from a cross-sectional survey to measure political, social, and ideological attitudes and self-reported behaviors related to the onset of the pandemic. The survey consisted of more than 70 main questions, with many using matrix tables. As a result, there were over 200 possible responses, and the majority used Likert scale responses to measure agreement with statements. Many of the questions utilized pre-existing scales, such as the just world scale (Lucas et al., 2011), the police legitimacy scale (Tankebe et al., 2016), and the authoritarianism scale measuring aggression, submission, and conventionalism (ASC) (Dunwoody & Funke, 2016).

Respondents had the option to provide an email address to enter a raffle to win one of 50 $10 gift cards. The survey and the study were approved by the Stockton University IRB. The survey was available online using Qualtrics for nearly two months, beginning in early June 2020 and ending in August. The authors distributed the survey through their own personal and professional networks via email and social media. Following the 2nd author’s institutional ethics approval, which covered data collection from US-based platforms, the links to the survey were also posted in the Facebook comments section of COVID-related videos or articles.

### 2.1. Sample

Survey fatigue and incomplete surveys were expected, given the length. While there were 2742 responses, cases with less than a 95 percent completion rate were removed from the dataset as well as those from outside of the United States, leaving 1262 total responses. The survey demographics (Table 1) indicate that most of respondents were white (81.38%), female (69.03%), and Democrat (49.8%). Participants’ ages ranged from 18 to 85 (*M* = 40.87, *SD* = 15.47). 71.15 percent of respondents held a bachelor’s degree or higher, and 81.54 percent reported that they live in an urban or suburban area, thus making the sample representative of the highly educated section of the US population rather than reflective of the ‘average citizen’.

### 2.2. Variables

In addition to gathering demographic data, this study used a variety of scales and composite variables as specific measures of attitudes about legitimacy, fear of COVID, and various levels of government (Table 2). All composite variables and scales reflected good or excellent reliability with a Cronbach’s alpha (a) of 0.78 or higher.

#### 2.2.1. Legitimacy Scale

To measure respondents’ perceptions of legitimacy for those they considered responsible for addressing the pandemic, the study utilized Tankebe et al.’s (2016) multi-dimensional model of legitimacy. Originally developed to measure police legitimacy, the scale encompasses four theoretically distinct dimensions: lawfulness, procedural fairness, distributive fairness, and effectiveness. The questions were adjusted to reflect the pandemic context (e.g., “I feel safe walking in my neighborhood at night” was changed to “I feel safer because of their actions”), and aimed to measure the degree to which respondents felt that the government, or the person they believed to be most responsible for addressing COVID-19, was acting in a manner that was right, just, and aligned with the will of the people. Participant responses were measured using a seven-point Likert scale ranging from 1 (*Strongly Disagree*) to 7 (*Strongly Agree*).

A composite legitimacy score was computed as the sum of all 16 items. Internal consistency was high (α = 0.988, average inter-item *r* = 0.836), though this should be interpreted with caution. Exploratory factor analysis indicated that the scale was empirically unidimensional in this sample, with a single factor accounting for 83.9% of the variance and an eigenvalue of 13.58, while no subsequent factor exceeded an eigenvalue of 0.52. Factor intercorrelations under an oblimin rotation further showed that the procedural and distributive fairness subscales were highly collinear (*r* = 0.95), suggesting that respondents evaluated all legitimacy dimensions cohesively rather than distinctly. This pattern likely reflects the highly polarised political context of data collection, wherein global approval or disapproval of a political actor tended to compress across all dimensions simultaneously. The high internal consistency therefore reflects item-level redundancy rather than exceptional measurement precision, and the four-subscale structure proposed by Tankebe et al. (2016) did not emerge empirically in this sample. Individual subscale reliabilities were as follows: procedural fairness (α = 0.985), distributive fairness (α = 0.968), and effectiveness (α = 0.921); lawfulness was assessed by a single item and could not be evaluated for reliability. These findings are consistent with empirical evidence that legitimacy judgments, particularly in politically charged contexts, exhibit high intercorrelations and limited discriminant validity, effectively collapsing into a single evaluative dimension (e.g., Gau, 2011; Jackson et al., 2012; Sunshine & Tyler, 2003).

#### 2.2.2. Fear of COVID-19

The composite variable, *fear of COVID*, was created using the sum of responses from 10 questions measuring participants’ attitudes or perceptions about the seriousness of the virus and the likelihood of contracting, suffering from, or dying as a result of COVID-19 (Table 3). Questions asked respondents if they believed it to be a serious public health issue, and if they feared that their friends or family may suffer from the virus. Responses were measured again using a seven-point Likert scale (a = 0.83).

#### 2.2.3. Perceptions of Government

Eight questions were used to assess perceptions of federal and state government and their response to the pandemic. The questions asked about the public health orders, the decision-making process, preparation for a pandemic, financial support, and general satisfaction with the governmental response. Responses were measured using a seven-point Likert scale, and two composite variables, *perceptions of federal government* (a = 0.88) and *perceptions of state government* (a = 0.89), were created using sums from the eight questions.

#### 2.2.4. Authoritarianism

Prior research has often found an empirical relationship between authoritarian views and support for Donald Trump (Choma & Hanoch, 2017; Conway & McFarland, 2019; Van Assche et al., 2019). Given that the items of the classical Right-Wing Authoritarianism (RWA) scale (Altemeyer, 1981) do not particularly align with the topic of our survey, we utilized sections of the Dunwoody and Funke (2016) Aggression–Submission–Conventionalism (ASC) scale, a multidimensional measure of authoritarianism based on Altemeyer’s original constructs. Given that the conventionalism scale created the same issues as the Altemeyer scale, only the submission (a = 0.77) and aggression (0.82) scales were used. Acknowledging the use of an incomplete tool, it was our joint team’s decision based on balancing the cost of limitation and maximizing survey completions, while reducing participant attrition, and based on adapting the tool to our specific research purposes. Questions from these scales measured attitudes about being critical of leaders, questioning authority, and encountering strong force by authorities. Scores from these two scales were used to create the composite variable, *authoritarianism* (a = 0.77).

#### 2.2.5. Political Ideology

Because this study aimed to understand the influence of political ideas as opposed to political party affiliation, respondents were offered a sliding scale from 1 (very liberal) to 7 (very conservative). This allowed for additional nuance of political attitudes beyond simple party identification, voter registration, etc.

#### 2.2.6. COVID Knowledge and Risk Factors

This study also posited that knowledge of or relationships with people who were hospitalized or died due to COVID-19 would affect attitudes about or fear of the virus. This dichotomous variable, *know others* (*n* = 1253), was supplemented with a question that asked about the nature of the relationship (Table 4). Of those who said they knew someone (*n* = 475), the most common responses were “distant,” such as a friend of a friend (*n* = 222) and “acquaintances” (*n* = 204). Other common responses included “someone in my extended family” (*n* = 119) and “a close friend” (*n* = 87).

Additionally, the survey asked if the respondents had any underlying health conditions that put them in the high-risk category for serious illness if they were infected with COVID-19 (*n* = 1253). To avoid intrusive questions about medical and health issues, this, too, offered a dichotomous yes/no variable, *COVID risk (self)*, as well as an option to respond “prefer not to answer” (Table 5).

#### 2.2.7. Change in Trust

To measure the impact that COVID-19 may have on perceptions of federal and state government, respondents were asked to rate their overall trust in government both before and after the pandemic. Using a seven-point Likert scale from “extremely untrustworthy” to “extremely trustworthy,” the variables, *change in federal trust* and *change in state trust*, reflected the difference between “after COVID” and “before COVID” (Table 6).

#### 2.2.8. Safe Behaviors

Engagement in COVID-19 protective behaviors was assessed using eight items drawn from two sections of the survey. Two items measured behavioral frequency using a five-point scale (1 = *Never*, 5 = *Always*): *wearing a mask in public* and *maintaining at least six feet of distance from strangers*. Six items measured agreement with protective behavioral intentions and practices using a seven-point Likert scale (1 = *Strongly Disagree*, 7 = *Strongly Agree*)*: taking early precautions before public health orders were mandatory, only leaving the house when absolutely necessary, avoiding physical interactions with non-household members, believing in individual responsibility to prevent the spread of COVID-19, intending to stay home until a vaccine was widely available*, and *hesitancy to be in large groups*. The two frequency items were rescaled to a 1–7 range prior to summation to ensure comparability across items. Internal consistency was acceptable (*α* = 0.881).

## 3. Results

First, to compare the mean legitimacy scores, an ANOVA was conducted with the individual or entity as the selection variable. There was a statistically significant difference in legitimacy scores based on whom respondents believed to be responsible for addressing COVID-19. Those who viewed the president or federal government as most responsible had lower legitimacy scores than those who reported their governor, state government, or local official or government to be responsible [*F*(5, 1219) = 135.71, *p* < 0.001].

Post hoc comparisons using the Tukey HSD test indicated that the differences in mean *legitimacy* scores were statistically significant for all comparisons to the President and the federal government. Those that were not significant were state government (*M* = 69.31, *SD* = 24.35) and governor (*M* = 72.81, *SD* = 27.39); governor and local government (*M* = 64.71, *SD* = 24.79); state government and local leader (*M* = 72.28, *SD* = 23.59); local leader and local government; and governor and local leader (Table 7).

Note, to address the potential confounding influence of party affiliation, we re-estimated the model with Democrat, Independent, Libertarian, and Other dummy variables added (Republican was used as the reference). The party block did not significantly improve model fit (Δ*R*^2^ = 0.005, *F*(4, 912) = 2.24, *p* = 0.063), and key coefficients changed negligibly (all Δ*β* < 0.04), confirming that the original specification was not meaningfully confounded by the omission of party affiliation. Next, to address the growing conspiracy theory that certain political groups or supporters may view the pandemic as a Democratic hoax, it was necessary to better understand and compare concern or fear about the pandemic based on political affiliation. Interestingly, those who responded “other” to the question about their political affiliation had the greatest average *fear of COVID* score. Respondents who aligned with the Republican party had the lowest mean for fear of COVID, while Democrats had the second highest. Predictably, independents, libertarians, and those with no political affiliation averaged between the two polar parties. A one-way between-subjects ANOVA was conducted, and there was a significant difference between political party groups based on *fear of COVID* [*F*(5, 1238) = 55.46, *p* < 0.001].

Post hoc comparisons using the Tukey HSD test indicated that the differences in mean scores for *fear of COVID* were statistically significant between nearly all political parties. Those that were not significant were Republican (*M* = 39.83, *SD* = 0.94) and Libertarian (*M* = 42.49, *SD* = 11.33); Democrat and Other (*M* = 52.17, *SD* = 7.61); Independent (*M* = 47.64, *SD* = 11.09) and No affiliation (*M* = 48.95, *SD* = 10.44).

To compare pre- and post-COVID levels of trust in both federal and state government, paired sample *t*-tests were conducted, particularly to highlight the temporal ordering for governmental attitudes to predict legitimacy. Although it would be reasonable to predict that those who were, for example, critical of the first Trump administration before COVID would be equally as critical during, the *t*-test results demonstrate that there was a statistically significant net negative change in trust for the federal government as a result of their responses and handling of the pandemic. The frequency distribution indicates that for 25.52 percent of respondents, their trust in the federal government decreased to varying degrees as a result of COVID-19, while it had no impact on trust for 515, or 40.80 percent of respondents.

Regarding the state government, the pandemic negatively impacted trust for 326 respondents (25.83 percent), but it incrementally increased trust for 192 respondents (15.21 percent). To determine the factors that would predict perceptions of the federal government’s response to the pandemic, the researchers used a multiple regression model. Table 8 presents the results of the regression, with *fear of COVID, authoritarianism, political ideology, age, and change in federal trust* all being significant predictors. This and all subsequent regression models were checked for issues of multicollinearity, and all VIF values were below 10, indicating no issues with variables being too highly correlated.

The total variance explained by this model was 48% (*R*^2^ = 0.48, *F*[7, 957] = 126.70), *p* < 0.001), and the model demonstrates that relationships with those who have been hospitalized or died as a result of COVID (knowledge of COVID patients) and individual risk factors for COVID (e.g., diabetes, immunosuppression, etc.) are not significant variables in predicting perceptions of the federal government’s handling of the pandemic. Instead, the most significant factors are *authoritarianism* (*ß* = 0.43, *p* < 0.001), *fear of COVID* (*ß*= −0.15, *p* < 0.001), *age* (*ß* = −0.11, *p* < 0.001), change in federal trust *(ß* = 0.19, *p* < 0.001), and *political ideology* (*ß* = 0.20, *p* < 0.001). Unsurprisingly, for the latter, those respondents whose political attitudes leaned more conservative were more likely to have a positive perception of the federal government.

Knowing that there were significant differences between groups of respondents, as evidenced by the earlier ANOVA, the researchers aimed to predict the legitimacy of the government representative or entity towards what respondents viewed as most responsible for addressing the pandemic. The purpose of this was to compare the influence of COVID-19 and political ideologies. Given the polar nature of modern politics, these models sought to compare similar variables and their influence on legitimacy. As such, four multiple regression models were conducted using the total sample before conducting separate models for the most common responses: the President, the federal government, and the state leader (Table 9). A Chow test confirmed that regression coefficients differed significantly across the four responsible-entity groups, *F*(12, 1042) = 76.11, *p* < 0.001, justifying the separate subgroup estimation approach. Using three variables—*federal trust change, political ideology, and fear of COVID*—these models demonstrated that although political affiliations were significant in predicting the legitimacy of the President, fear of COVID-19 was the only significant factor in all four models. While political ideology predicted respondents’ perceptions of legitimacy for the president and federal government, it was not significant in predicting legitimacy when they believed that the state governor was the primary entity or authority responsible for addressing the pandemic. A model was also estimated for respondents who attributed responsibility to the state government (*N* = 179), which was significant overall, *F*(3, 175) = 7.54, *p* < 0.001, *R*^2^ = 0.114. Consistent with the Governor model, fear of COVID was a significant predictor (*B* = 0.509, *p* = 0.007) and political ideology was significant but in the negative direction (*B* = −3.28, *p* = 0.015), while change in federal trust was non-significant (*p* = 0.965), consistent with the pattern observed for state-level attribution more broadly.

Note, extended legitimacy models incorporating the full predictor set from Table 8 (authoritarianism, age, know others, COVID risk) were also estimated for the total sample and each subgroup (see Table 10). In the total sample, adding these predictors increased *R*^2^ from 0.132 to 0.210 (Δ*R*^2^ = 0.078, *F*[4, 909] = 22.54, *p* < 0.001). Authoritarianism was shown as the primary additional predictor across models (*β* = 0.335, *p* < 0.001; President subgroup: *β* = 0.446, *p* < 0.001). In addition, fear of COVID was non-significant in the total sample extended model (*β* = −0.043, *p* = 0.226) once authoritarianism was included.

Finally, to measure the ways in which legitimacy and other factors affect safe behaviors and individual decisions to engage in protection measures during the pandemic, multiple regression models were used, comparing the effects of five variables—*legitimacy, fear of COVID, political ideology, COVID risk (self)*, and *know others* (Table 11). Separating between groups that view various entities and individuals responsible (to address differences in legitimacy), Table 10 compares the total sample with the groups who view the President, federal government, and governor responsible, respectively. *Legitimacy* was a significant variable in predicting safe behaviors for two groups: the President (*ß*= −0.18, *p* = 0.001) and the governor (*ß*= −0.78, *p* < 0.001), though in opposite directions. Decreased legitimacy in the President predicted a decreased likelihood of safe behaviors, whereas increased legitimacy in a respondent’s governor increased the likelihood of safe behaviors. *Political ideology* was only a significant factor in the overall model (*ß*= −0.08, *p* = 0.004) and the federal government model (*ß*= −0.18, *p* < 0.001), indicating that the legitimacy of the President has more of an effect on safe behavior than does political ideology.

The only variable that was consistently significant across all models was *fear of COVID*. This indicates that regardless of whom the respondents believed to be responsible for addressing the pandemic, their overall fear of the pandemic affected their behavior. This is particularly interesting, however, given that there appears to be no impact of individual risk factors or knowledge of others who have suffered and/or died as a result of the disease. Instead, simply the belief that COVID is a serious public health issue and the fear of it harming oneself or others outweighs the actual instances of the disease affecting them or people they know.

## 4. Discussion

In this study, we examined individual- and group-level psychological factors associated with perceptions of the legitimacy of government restrictions during the COVID-19 pandemic, as well as intentions to engage in safe behaviors among US citizens. First, we found clear differences in perceived threat from COVID-19 across political affiliations. Individuals who aligned with the Republican Party reported the lowest levels of fear of COVID-19, whereas Democrats reported the second-highest levels. This finding is consistent with previous research showing that political conservatives perceived COVID-19 as a significantly lower threat (Christensen et al., 2020; Hsiehchen et al., 2020). Importantly, political affiliation has also been shown to strongly influence compliance behavior during the pandemic, regardless of party orientation. For example, Gadarian et al. (2021) demonstrated in a large US-based survey that partisanship was the strongest predictor of compliance with COVID-19 policies. Several additional studies similarly identify political identity as a primary determinant of pandemic-related attitudes and behaviors (e.g., Collins et al., 2021; Gadarian et al., 2021; Makridis & Rothwell, 2020; Raile et al., 2024).

Second, our results indicate that the pandemic was associated with decreased trust in both the federal and state governments. This finding contrasts with research conducted in other national contexts. For instance, Esaiasson et al. (2021), using a large web-based survey of over 10,000 Swedish adults, found that the COVID-19 crisis led to increased institutional trust. One possible explanation for this divergence lies in differences in governance approaches. In Sweden, pandemic management relied largely on voluntary cooperation rather than legally enforced restrictions (Kuhlmann et al., 2021; Larsson, 2022), which may have fostered trust rather than eroded it (Bengtsson & Brommesson, 2022).

Prior research has shown that trust in a government’s intentions and capacity can promote willing compliance during crises (Taylor et al., 2009). From a theoretical perspective, these findings can be interpreted through the lens of social identity theory (Tajfel, 1982). During times of crisis, individuals may experience an increased need to affiliate with trusted in-group members and institutions. This dynamic can result in short-term surges in support for political leaders or governments, a phenomenon often referred to as the “rally effect.” Such effects may also be amplified by heightened emotional states, including fear and anxiety, during periods of uncertainty (Lambert et al., 2011).

Interestingly, we also found that participants who attributed primary responsibility for pandemic management to the president or the federal government reported lower legitimacy ratings than those who identified their governor or state government as most responsible. This finding aligns with research by Grossman et al. (2020), who showed that political partisanship shaped individuals’ willingness to voluntarily comply with COVID-19 mitigation measures in response to communications. Notably, these effects were stronger in Democratic-leaning counties, which also responded more positively to recommendations from Republican governors than from Democratic governors. Grossman et al. (2020) suggested that these patterns may have emerged partly due to inconsistent messaging, particularly in Republican-led states where some governors emphasized the seriousness of COVID-19 while the US president at the time expressed skepticism about its impact.

Our findings are also consistent with prior work showing that partisan identity structures attitudes toward governmental legitimacy across different federal levels (Jacobs, 2021). Democrats and Republicans may assign divergent responsibilities to local, state, and federal governments, leading to disagreement about which authority is accountable for crisis management. Such divergence may generate mixed messages and confusion regarding responsibility, thereby undermining institutional trust. Supporting this interpretation, Arceneaux et al. (2020) found that support for policies that infringe on civil liberties during crises was more likely when those policies were endorsed by in-group political actors and trusted experts. When messages from state and federal authorities are misaligned or their roles are perceived as unclear, in-group trust and loyalty may weaken, reducing perceived legitimacy.

While the data were collected a few years ago and may be seen as ‘somewhat dated’, our findings may help inform policy makers and researchers dealing with yet-to-come crises involving massive uncertainty and fear (e.g., Besta et al., 2025; Palace et al., 2024), helping them understand and respond to political attitudes. Classical research within the psychometric paradigm (Slovic, 1987) has consistently demonstrated that public responses to risk are not reducible to deficits in knowledge but instead reflect qualitatively different evaluative frameworks grounded in values and affect. Within this perspective, while group-level factors such as political trust and responsibility attribution remain important in shaping perceptions of institutional legitimacy, they do not operate in isolation. Rather, individual-level emotional responses, particularly fear, play a central role in how risks are perceived. Fear functions as an affective heuristic, enabling individuals to rapidly assess the severity and acceptability of a risk without reliance on technical calculations of probability. Consequently, risks that evoke high levels of dread or perceived lack of control are more likely to be judged as unacceptable, regardless of their statistical likelihood, and may simultaneously erode trust in governing institutions. This helps explain why public attitudes and legitimacy judgments can diverge from expert assessments even in contexts of high information availability: individuals are not merely interpreting risk cognitively but are engaging in emotionally grounded evaluations shaped by concerns about control, fairness, and potential harm. In this sense, affective responses such as fear may be as influential as, or even more influential than, institutional factors in determining both perceived legitimacy and the adoption of safe behavior.

Taken together, our findings suggest that the legitimacy of pandemic restrictions depends not only on the substance of policies but also on how responsibility is perceived within a federal system. When responsibility is attributed to national political actors, where pandemic governance became highly polarized, evaluations of restrictions are more likely to be filtered through partisan and blame-oriented lenses, resulting in lower perceived legitimacy. In contrast, attributing responsibility to governors or state governments may localize accountability and mitigate the legitimacy costs associated with national-level politicization.

At the interpersonal level, we found that authoritarian personality traits were positively related to perceived legitimacy of restrictions. This result aligns with prior work by Peng (2022), who reported nuanced but consistent effects of authoritarianism on support for restrictive policies during the pandemic. In addition, across all analytical models, fear of COVID-19 emerged as the strongest predictor of both perceived legitimacy and intentions to engage in safe behavior. Regardless of macro-level factors such as political trust or responsibility attribution, individuals’ fear of COVID-19 consistently motivated compliance-related attitudes and behaviors. This finding corroborates previous research highlighting the central role of emotions, particularly fear, in shaping pandemic responses (Liu & Yang, 2023; Vasilopoulos et al., 2023).

Several limitations should be acknowledged, including the reductionist nature of some questions. For example, anticipating participant fatigue and attrition, we chose to rely on some composite scales rather than on more distinct ones (e.g., personal and altruistic fear). Relatedly, the question about the person or entity most responsible for creating regulations to address the pandemic is under-nuanced and likely deserves a separate, deeper study involving a series of more sensitive questions (e.g., about the presidential office in general, a particular president, government makeup and one’s political interests, to name just a few). Some of these new questions would likely benefit from qualitative exploration. Further, while the adapted Tankebe et al. (2016) legitimacy scale demonstrated strong internal consistency (α = 0.988), exploratory factor analysis indicated that the four-subscale structure (lawfulness, procedural fairness, distributive fairness, and effectiveness) did not emerge empirically, with a single factor accounting for 83.9% of variance. This likely reflects the politically polarised context of data collection, in which evaluations of all legitimacy dimensions collapsed into a single global judgment, rather than a failure of the instrument. The scale was not formally validated for pandemic governance contexts, and future research should conduct confirmatory factor analysis and consider reporting subscale scores separately to preserve the theoretical distinctions the instrument was designed to capture.

Importantly, all measures were based on self-reports, which, despite anonymity, may be subject to bias and may not fully capture actual behavior. Additionally, attitudes and behaviors may have evolved over the course of the pandemic, limiting the temporal generalizability of our findings that cannot be extended to the ‘ordinary US citizen’. While our recruitment was aimed at the broad American public, it is likely that those most willing to take part in the survey were the ones who found the topic of the COVID-19 pandemic and political attitudes most personally relevant, thus resulting in a sample not reflective of the general US population. Furthermore, this issue was probably compounded by the recruitment strategy involving social media, whose contents amplified the threats associated with the pandemic.

The composition of the sample may also have shaped the relative strength of the observed predictors, particularly the finding that fear of COVID-19 emerged as the dominant predictor of perceived legitimacy. The overrepresentation of Democratic-leaning and highly educated respondents may have amplified the association between fear and legitimacy. Prior work (Chykina & Crabtree, 2023; Camacho-García et al., 2025) shows that these groups report greater perceived pandemic threat and stronger support for mitigation measures, which may have restricted ideological variance toward respondents already predisposed to view COVID-19 as a serious risk. In a more politically and socioeconomically representative sample, this relationship would likely remain present but may be attenuated by greater inclusion of individuals with lower threat sensitivity, weaker institutional trust, or stronger reactance toward restrictions. Accordingly, the apparent dominance of fear in the present models should be interpreted as contingent, at least in part, on the attitudinal profile of the sampled population.

It is recommended that future similar studies consider incentivizing participants in monetary terms and using platforms dissociated from news contents. It also must be noted that the data were collected between June and August 2020, a period spanning the initial lockdown phase, the beginning of easing restrictions, and the emergence of major protests following the killing of George Floyd. While our work did not account for such temporal variation, we highlight the need for the inclusion of time-trend variables in follow-up research.

As the study was conducted exclusively in the United States, results may differ substantially in other national contexts with different political systems, governmental strategies, and communication styles. As demonstrated by the Swedish case, governance structures and messaging approaches can fundamentally alter public responses. Future research should thus adopt comparative designs to examine how governmental structure, political communication, and psychological factors interact across countries.

## Figures and Tables

**Table 1:** Demographic Statistics.

**Age**	
Mean (*SD*)	40.87 (15.47)
Min–Max	18–85
**Gender (%)**	
Male	342 (27.29)
Female	865 (69.03)
Other	46 (3.67)
**Race (%)**	
White	1027 (82.29)
Black	33 (2.64)
Asian	34 (2.72)
Two or more	51 (4.09)
Other	117 (9.38)
**Political Party (%)**	
Republican	166 (13.27)
Democrat	628 (50.20)
Independent	220 (17.59)
Libertarian	67 (5.63)
Tea Party	1 (0.08)
Other	44 (3.52)
No affiliation	125 (9.99)

Numbers may not add to 100% due to missing responses.

**Table 2:** Person or Entity Most Responsible for Creating Regulations to Address the COVID-19 Pandemic.

	Total	Percent
President	440	35.31
Federal Government	379	30.42
State Governor	181	14.53
State Government	200	16.05
Local Leader	19	1.52
Local Government	36	2.89

**Table 3:** Descriptive Statistics for Independent and Dependent Variables.

	Min	Max	a	Mean (*SD*)
Legitimacy	16	112	0.99	46.19 (30.13)
Fear of COVID	10	70	0.83	48.93 (10.45)
Perceptions of Federal Govt	8	56	0.87	23.25 (10.21)
Perceptions of State Govt	8	56	0.88	34.33 (10.86)
Authoritarianism	12	68	0.84	34.58 (11.17)
Change in Federal Trust	−5	3		−0.86 (1.08)
Change in State Trust	−5	5		−0.17 (1.20)

**Table 4:** Respondents’ Personal Knowledge of COVID Victims.

	Frequency	Percent
Yes	475	37.6
No	771	61.1
Prefer not to answer	13	1.0

**Table 5:** Respondents’ Underlying Health Conditions.

	Frequency	Percent
Yes	472	37.4
No	746	59.1
Prefer not to answer	35	2.8

**Table 6:** Correlation Matrix.

	1	2	3	4	5	6	7	8	9	10
1. Legitimacy	-									
2. Fear of COVID	−0.20 **	-								
3. Perceptions of Federal Govt	0.52 **	−0.37 **	-							
4. Perceptions of State Govt	0.22 **	0.17 **	0.13 **	-						
5. Authoritarianism	0.46 **	−0.34 **	0.60 **	0.10 **	-					
6. Political Ideology	0.33 **	−0.43 **	0.50 **	−0.08 **	0.55 **	-				
7. Know others	−0.05	0.13 **	−0.06 *	0.04	−0.05	−0.05	-			
8. COVID risk (self)	−0.05	0.28 **	−0.06 *	0.01	0.00	−0.05	0.11 **	-		
9. Change in Federal Trust	0.13 **	−0.12 **	0.28 **	0.01	0.10 **	0.09	−0.05	0.09 **	-	
10. Change in State Trust	0.06 *	0.13 **	−0.06 *	0.51 **	−0.05	−0.12 **	0.02	0.00	0.17 **	-

* *p* < 0.05; ** *p* < 0.01.

**Table 7:** Mean Comparison of Legitimacy Scores.

	*N*	Min	Max	Mean	*SD*	Std Error
President	432	16	112	30.72	25.23	1.21
Federal Government	366	16	106	35.86	21.03	1.10
Governor	177	16	112	72.81	27.39	2.06
State Government	198	16	112	69.31	24.35	1.73
Local leader	18	16	110	72.28	23.59	5.56
Local government	34	16	112	64.71	23.79	4.24

*F* = 135.71, *p* < 0.001.

**Table 8:** Predicting Perceptions of Federal Government Response to the Pandemic.

Variable	B	SE	*ß*	t
Constant	15.51 ***	1.86		8.36
Authoritarianism	0.38 ***	0.03	0.43	14.91
Fear of COVID	−0.15 ***	0.03	−0.15	−5.55
Political Ideology	1.31 ***	0.19	0.20	6.87
Age	−0.07 ***	0.02	−0.11	−4.54
Know others	0.02	0.46	0.00	0.04
COVID risk (self)	0.45	0.46	0.03	0.98
Change in federal trust	1.79 ***	0.22	0.19	8.24

*** *p* < 0.001.

**Table 9:** Comparative Models Predicting Legitimacy of Perceived Authorities who Addressed the Pandemic.

Legitimacy Prediction	B	SE	*ß*	*t*	*F* (df)	*R* ^2^
**Total**					51.90 (3, 1105) ***	0.12
Constant	40.11 ***	5.66		7.09		
Federal trust change	1.99 ***	0.79	0.72	2.52		
Political ideology	5.63 ***	0.61	0.29	9.22		
Fear of COVID	−0.24 **	0.90	−0.08	−2.65		
**President**					108.47 (3, 393) ***	0.46
Constant	34.72 ***	6.56		5.29		
Federal trust change	3.55 ***	0.84	0.16	4.23		
Political ideology	8.69 ***	0.62	0.54	13.62		
Fear of COVID	−0.54 ***	0.11	−0.20	−5.06		
**Federal Government**					32.09 (3, 344) ***	0.22
Constant	49.54 ***	7.85		6.21		
Federal trust change	1.39	0.98	0.07	1.42		
Political ideology	4.85 ***	0.83	0.31	5.84		
Fear of COVID	−0.53 ***	0.13	−0.23	−4.17		
**State Government**					11.64 (3, 171) ***	0.113
Constant	54.16	11.502		4.71		
Federal trust change	−0.927	1.820	−0.037	−0.51		
Political ideology	−2.751 *	1.335	−0.156	−2.06		
Fear of COVID	0.537 **	0.185	0.231	2.90		
**Governor**					11.64 (3, 143) ***	0.20
Constant	39.66 **	12.91		3.07		
Federal trust change	1.78	1.81	0.08	0.99		
Political ideology	−2.09	1.50	−0.12	−1.40		
Fear of COVID	0.91 ***	0.20	0.39	4.55		

* *p* < 0.05, ** *p* < 0.01, *** *p* < 0.0001.

**Table 10:** Extended Legitimacy Regression Models with Full Predictor Set.

	Total Sample	President
	*N* = 917	*N* = 312
Variable	B (*β*)	B (*β*)
*Constant*	17.742	18.878 **
*Change in Federal Trust*	1.725 (0.062) *	3.663 (0.171) ***
*Political Ideology*	2.522 (0.129) ***	4.441 (0.292) ***
*Fear of COVID-19*	−0.125 (−0.043)	−0.566 (−0.213) ***
*Authoritarianism*	0.912 (0.335) ***	0.964 (0.446) ***
*Age*	−0.127 (−0.064) *	−0.154 (−0.099) **
*Know Others*	−0.462 (−0.008)	−1.181 (−0.024)
*COVID Risk (Self)*	−1.342 (−0.022)	3.843 (0.079) *
*R*^2^	0.210	0.620
Adjusted *R*^2^	0.204	0.611
*F*	34.59 ***	70.83 ***
Δ*R*^2^ (vs. 3-predictor model)	0.078 ***	0.143 ***

* *p* < 0.05, ** *p* < 0.01, *** *p* < 0.0001. Note. Extended models for the Federal Government (*N* = 304) and Governor (*N* = 128) subgroups produced unstable estimates due to limited sample sizes relative to the number of predictors and are excluded from this table.

**Table 11:** Comparative Models Predicting Safe Behaviors, Selected on Perceived Authority of Addressing the Pandemic.

Behavior Prediction	B	SE	*ß*	*T*	*F* (df)	*R* ^2^
**Total**					206.26 (5, 1096) ***	0.49
Constant	9.10 ***	1.05		8.65		
Legitimacy	−0.00	0.01	−0.02	−0.70		
Fear of COVID	0.42 ***	0.02	0.66	25.79		
Political ideology	−0.32 **	0.11	−0.08	−2.90		
COVID risk (self)	−0.20	0.28	0.02	0.70		
Know others	0.36	0.29	−0.03	−1.26		
**President**					48.01 (5, 393) ***	0.38
Constant	14.11 ***	1.78		7.95		
Legitimacy	−0.04 **	0.01	−0.18	−3.43		
Fear of COVID	0.33 ***	0.03	0.54	11.45		
Political ideology	0.23	0.19	0.06	1.20		
COVID risk (self)	−0.03	0.79	−0.00	−0.03		
Know others	−0.98	0.88	−0.61	−1.11		
**Federal Government**					52.40 (3, 342) ***	0.44
Constant	12.89 ***	2.08	6.20	6.21		
Legitimacy	00.01	0.01	−0.02	−0.37		
Fear of COVID	0.37 ***	0.03	0.55	11.06		
Political ideology	−0.78 ***	0.21	−0.18	03.68		
COVID risk (self)	0.718	0.47	0.07	1.54		
Know others	−0.57	0.49	−0.49	−1.16		
**Governor**					44.31 (3, 139) ***	0.62
Constant	1.76	2.63		0.67		
Legitimacy	0.07 ***	0.02	0.26	4.29		
Fear of COVID	0.43 ***	0.04	0.69	9.78		
Political ideology	0.31	0.29	0.65	1.07		
COVID risk (self)	−0.03	0.79	−0.00	−0.31		
Know others	−0.98	0.88	−0.05	−1.11		

** *p* < 0.01, *** *p* < 0.0001.

## Data Availability

The raw data supporting the conclusions of this article will be made available by the authors upon request.
